# Acquired Von Willebrand Factor Deficiency at Patient-Prosthesis Mismatch after AVR Procedure—A Narrative Review

**DOI:** 10.3390/medicina59050954

**Published:** 2023-05-16

**Authors:** Andrei Emanuel Grigorescu, Andrei Anghel, Darius Gheorghe Buriman, Horea Feier

**Affiliations:** 1 Department of Cardiology, “Victor Babes” University of Medicine and Pharmacy Timisoara, 300041 Timisoara, Romania; grigorescu.andrei@umft.ro (A.E.G.);; 2 Research Center of the Institute of Cardiovascular and Heart Disease of Timisoara, 300310 Timisoara, Romania; 3 Division of Cardiovascular Surgery, Institute for Cardiovascular Diseases, 300391 Timisoara, Romania; 4 Doctoral School Medicine—Pharmacy, “Victor Babes” University of Medicine and Pharmacy Timisoara, 300041 Timisoara, Romania; 5 Department of Biochemistry, “Victor Babes” University of Medicine and Pharmacy Timisoara, 300041 Timisoara, Romania; 6 Center for Translational Research and Systems Medicine, “Victor Babes” University of Medicine and Pharmacy Timisoara, 300041 Timisoara, Romania

**Keywords:** acquired, von Willebrand factor, deficiency, aortic stenosis, patient-prosthesis mismatch

## Abstract

Acquired von Willebrand factor deficiency has been described in patients with aortic valve stenosis due to high shear forces developed during passage through the narrowed valve orifice, which determines structural changes in this molecule. Similar flow conditions are present in patients with an aortic prosthesis that presents a patient-prosthesis mismatch. Patient-prosthesis mismatch is described by the smaller effective orifice area of the prosthesis than the native valve, which would probably determine similar changes in the molecules of the von Willebrand factor, leading to acquiring von Willebrand deficiency.

## 1. Data Acquisition

To investigate acquired von Willebrand Factor Deficiency (AVWFD) at the patient-prosthesis mismatch (PPM) after aortic valve replacement (AVR), we conducted an extensive literature search and review using a variety of search engines and sources, including MDPI, PubMed, Google Scholar, and Springer. The search was performed using the keywords “aortic stenosis”, “patient-prosthesis mismatch”, “acquired von Willebrand deficiency”, and “aortic valve replacement.” We did not impose any year limitation on the database search, and we collected additional articles by reviewing the references of the screened articles. According to the criteria, 35 bibliographic sources related to the studied topic were eligible for this review article.

To collect information on study design, patient demographics, AVR procedure characteristics, AVWFD diagnosis, and patient outcomes, we used a standardized data extraction form. We included in extenso published articles, books, and papers that reported AVWFD at PPM after the AVR procedure. The data were synthesized and summarized in a narrative format, highlighting the main findings and trends across the literature.

The narrative review aimed to provide a comprehensive overview of the current understanding of AVWD at PPM after the AVR procedure. This condition is not well understood, and its diagnosis and management pose a significant clinical challenge. Our review identified the need for a standardized approach to diagnosing and managing AVWFD, as well as the need for more research to understand the underlying mechanisms of the condition.

In addition, our review revealed conflicting findings on the impact of PPM on patient outcomes, including long-term survival. While some studies reported a significant impact of PPM on outcomes, others did not find any significant association. This suggests the need for more robust studies with larger sample sizes and longer follow-up periods to clarify the impact of PPM on patient outcomes.

Overall, our data acquisition process involved a thorough and systematic approach to identifying relevant literature on AVWFD at PPM after the AVR procedure. The data were synthesized and summarized in a narrative format to provide a comprehensive overview of the current understanding of this condition. Our review highlights the need for standardized diagnostic and management approaches and more research to understand the mechanisms underlying AVWFD.

## 2. AVR Procedure

Aortic stenosis is the most frequent valvulopathy in Europe and North America [1] and the most common indication for aortic valve replacement surgery. Most frequently, aortic stenosis is diagnosed after the age of 65 years; its prevalence is 4–5% in the general population aged over 65.

Aortic stenosis can occur in tricuspid valves, as well as in congenitally bicuspid valves, in a gradual process of leaflet thickening and calcification that extends to the aortic annulus and root. The progress of the calcification process will determine, in the end, a small stenotic-resistant orifice through the almost immobile cusps. Symptoms may develop at this stage in the form of dyspnoea, chest pain, and syncope, but they may be absent, and the disease may be diagnosed only once sudden death occurs.

Imaging techniques such as ultrasonography, cardiac catheter exam, CT, or MRI quantify the severity of the disease by measuring the aortic orifice or the gradients across the stenotic valve. The aortic valve stenosis is quantified secondary to the transthoracic cardiac ultrasound, which remains the main method to assess aortic stenosis severity. By measuring and correlating the three standardized parameters—the peak velocity, the mean pressure gradient, and the aortic valve area—the aortic stenosis is graded as mild, moderate and severe, as presented in Table 1 [2].

Patients with severe aortic stenosis require aortic valve replacement (AVR). While this has been done exclusively by heart surgery until recently, Transcatheter Aortic Valve Replacement (TAVR) has become a valid alternative to open-heart surgery, particularly in elderly patients with comorbid conditions.

The choice of AVR procedure depends on various factors, including the patient’s age, overall health, and anatomy, as well as the severity and type of valve disease. AVR can provide significant improvement in heart function and quality of life for individuals with aortic valve disease, but it is associated with a number of potential complications, including bleeding, infection, and patient-prosthesis mismatch.

The full open surgical procedure for aortic valve replacement is a complex procedure that involves the following steps. First, the patient is given general anesthesia to ensure they are unconscious and pain-free during the surgery. Then, a long incision is made along the sternum. The sternum is divided, and the pericardium is exposed. For such types of procedures, cardiopulmonary bypass is obligatory. Therefore, the surgeon will insert two cannulas: one two-staged cannula into the inferior vena cava through the right atrial appendix to drain the blood to the CPB machine, and the other cannula is placed into the ascending aorta to return oxygenated blood from the CPB machine. During the CPB procedure, the patient’s vital signs are closely monitored to ensure that the heart and lungs are being supported properly. In addition, the surgeon and perfusionist (the specialist who manages the CPB machine) will monitor the patient’s blood pressure, heart rate, oxygen levels, and other parameters.

The aorta is temporarily clamped to stop blood flow so that the surgeon can replace the valve. The diseased aortic valve is then inspected and removed by excising it away from the aortic root. Once the old valve has been removed, the surgeon will need to replace it with a new one.

Once the annulus has been measured, the surgeon can select the appropriate prosthesis size. This may involve choosing a mechanical or bioprosthetic valve and selecting the specific model and size based on the patient’s individual needs.

The new valve is sewn into place in the aortic root. The sutures are carefully placed to ensure a tight seal around the valve, and the surgeon will check to make sure there are no leaks before removing the aortic clamp and restoring blood flow to the heart.

The chest is then closed using wires or surgical staples, and the incision is covered with dressings. After the procedure is complete, the patient is closely monitored in the intensive care unit for several days to ensure that the heart is functioning properly and that there are no complications.

## 3. Patient-Prosthesis Mismatch (PPM): Prevalence, Understanding and Avoidance

Aortic valve replacement is the most common cardiac surgery procedure; its goal is to replace the stenotic diseased valve with a competent and non-stenotic prosthesis [3].

One of the key considerations in aortic valve replacement procedures for patients with a small aortic root and a large body surface area is selecting the most appropriate prosthesis size. This is an important decision that can have a significant impact on patient outcomes. One of the potential complications that can arise from AVR procedures is a patient-prosthesis mismatch, which occurs when the size of the artificial valve is too small compared to the patient’s BSA and cardiac output.

PPM is a significant concern in AVR procedures as it can lead to suboptimal hemodynamics, increased shear stress on the valve, and a greater risk of acquired von Willebrand factor deficiency. In addition, when the artificial valve is too small, it can create a turbulent blood flow that can damage red blood cells and lead to bleeding.

The risk of PPM is particularly high in patients with a small aortic root and a large body surface area, as there is a limited range of prosthesis sizes available that can adequately fit their anatomy. Therefore, surgeons must carefully consider the available options and weigh the risks and benefits of each choice.

Patient-prosthesis mismatch was first evidenced by Rahimtoola in 1978, describing it as the situation when the effective orifice area (EOA) of the newly implanted valve is smaller than that of the native stenotic valve [4,5,6]. During the last decades, literature estimates the prevalence of moderate PPM to range between 20% and 70% and severe PPM between 2% and 10% [7].

The risk of PPM can be influenced by several factors, including patient anatomy, the size and type of artificial valve used, and the surgical technique employed. While factors that could determine a higher risk for developing PPM are not well established yet, several studies concluded that the female gender is at higher risk for PPM. This may be due to a smaller aortic annulus and the fact that they are older at the time of the procedure and, therefore, more likely to receive a biological prosthesis [8,9].

Another factor could be the aortic root diameter at the sinotubular junction, which frequently has a smaller diameter than the aortic annulus, especially in female patients, and obstructs the lowering of the prosthesis into the aortic annulus position. This situation constrains the surgeon to evaluate the greater good: decide to implant a smaller prosthesis and risk PPM, or decide to prolong the cross-clamp time by proceeding to an aortic root enlargement procedure in order to implant an optimal-sized prosthesis, knowing that this increases operative mortality [8,10,11].

Although the geometric orifice area is currently often used as a reference parameter when choosing the optimal prosthesis size, the effective orifice area is a more reliable one [3].

**Figure 1:**
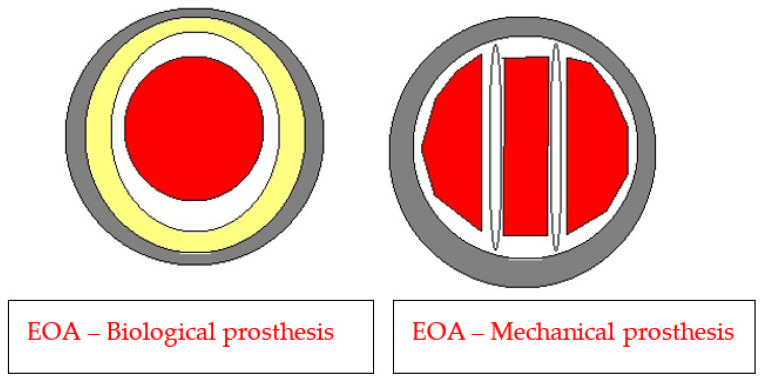
Understanding EOA Modified and adapted from Pibarot and Dumesnil [4].

Bioprosthetic valves usually have a smaller diameter and smaller EOAs when compared with mechanical prostheses or stentless prostheses, as presented in Table 2 and Figure 1 [4,8].

Understanding PPM is important for optimizing patient outcomes after AVR procedures. Echocardiography and computed tomography (CT) scans can be used to assess PPM and determine the degree of mismatch.

Focused postoperative cardiac ultrasonography assesses the patients after the aortic valve replacement procedure. Replacing the stenotic valve with a small prosthetic valve in relation to the patient’s body surface area (BSA) will lead to a lower EOA and higher transprosthetic pressure gradients, resulting, in effect, in a functional stenosis in the aortic outflow tract.

Transprosthetic pressure gradients measured postoperatively are related to EOA but also to the transprosthetic flow, which is related to cardiac output, which in turn, at rest is primarily related to the patient’s body surface area and left-ventricle systolic function. As a consequence, the only parameter that has been shown to be relevant to characterize patient-prosthesis mismatch would be the indexed EOA. The indexed EOA can be defined as the effective orifice area of the prosthesis divided by the patient’s body surface area (EOA/BSA). Rahimtoola, as well as Pibarot and colleagues, proposed PPM to be evaluated more precisely relating to the indexed EOA of a prosthesis into mild (>0.85 cm^2^/m^2^), moderate (0.65–0.85 cm^2^/m^2^) and severe (<0.65 cm^2^/m^2^) [3,4].

Avoiding PPM can be achieved through careful preoperative planning and assessment, including accurate measurement of the native annulus and the selection of an appropriate size of the artificial valve. Therefore in order to avoid PPM, the selection of optimal prosthesis size should be according to the target of iEOA [3,9]. As a further matter, Pibarot and Dumesnil [9] suggested an algorithm in order to avoid PPM: (1) calculation of the patient’s BSA; (2) determine the minimal prosthesis EOA required to ensure optimal iEOA; (3) choose a prosthesis in accordance with targeted iEOA. Finally, surgical strategies such as enlargement of the aortic root or use of stentless bioprosthesis should stand up for avoidance of PPM [3].

PPM is a common occurrence after AVR, but its impact on long-term survival remains controversial. Severe PPM has been associated with decreased long-term survival in some studies, but other studies have found no significant association [13,14,15]. Therefore, close monitoring of patients after AVR procedures is crucial to identify PPM and address any potential complications, including AVWFD.

## 4. Von Willebrand Factor Deficiency—Diagnostic Approach

The Von Willebrand factor deficiency can be inherited or acquired. In addition, genetic testing can identify mutations in the VWF gene and other genes involved in the coagulation cascade.

The von Willebrand factor (VWF) is a large adhesive and multimeric plasma glycoprotein that found its historical origin in 1924 [16]. It is synthesized in megakaryocytes and endothelial cells and is involved in platelet adhesion and blood clotting [17].

VWF multimers are vulnerable to physiologic degradation by the action of a certain metalloprotease named ADAMTS13 (A Disintegrin-like And Metalloprotease domain with Thrombospondin type I motif), its susceptibility being increased for type 2A form of von Willebrand disease.

Factors that could alter VWF plasma levels include age, race, ABO blood groups, inflammatory mediators, endocrine hormones, pregnancy, hypothyroidism, ageing and acute stress or inflammation [18].

The VWF gene is located on the short arm of chromosome 12—12p13.2, and a partially unprocessed pseudogene on VWF is located on chromosome 22q11.2 [19]. After analyzing the amino acid sequence, four distinct domains, each domain corresponding to a certain region of VWF [16].

Hemostasis is influenced by the contest between the biosynthesis of large VWF multimers and their degradation by ADAMTS13 metalloprotease. Low activity of ADAMTS13 can cause thrombotic thrombocytopenic purpura, whereas mutations in the A2 domain can lead to selective depletion of large VWF multimers, as found in VWD type 2A [16].

The von Willebrand Factor gene is complex and challenging for sequencing and interpretation. Therefore, genetic testing for von Willebrand disease may be recommended in certain cases to manage the patient’s condition. It may not be necessary for everyone, but it can provide useful information in specific situations [17].

Congenital Von Willebrand deficiency is a common inherited bleeding disorder, its prevalence of symptomatic individuals being ~1 in 10,000 [12]. Abnormalities in VWF affect normal hemostasis, as described since the 1950s [16,20,21].

Laboratory testing is a crucial part of diagnosing VWF deficiency and involves measuring VWF levels and activity in the blood. The main components in diagnosing vWD involve quantitative and qualitative measurements of vWF and FVIII: Factor VIII coagulant activity (FVIII:C)—measures functional activity of factor VIII; VWF antigen (VWF:Ag)—measures the amount of VWF; Ristocetin co-factor (VWF:RCo)—measures the functional activity of VWF [22].

Von Willebrand deficiency is categorized into three types, which are determined based on the nature of the deficiency. Type 1 and Type 3 denote a quantitative deficiency of VWF, while Type 2 denotes a qualitative deficiency of VWF.

As presented in Table 3, type 1 is the most prevalent type, characterized by lower VWF:Ag and VWF:RCo, and normal or borderline FVIII:C. Diagnosis of Type 1 is relevant when VWF:Rco/VWF:Ag ratio is >0.6. Type 3 VWD reveals very low or undetectable VWF:Ag and VWF:RCo, very low FVIII:C and absent VWF multimers. Type 2 VWD variants (2A, 2B, 2M, 2N) are characterized by different levels in VWF activity and different combinations of VWF:Rco/VWF:Ag ratios and high molecular weight multimers distribution [23].

In terms of providing further insight into von Willebrand disease, the U.S. National Institutes of Health publication no.08-5832/2007 served as a valuable resource by offering additional information. It covered the genetic inheritance and likelihood of passing on the disease to offspring, as well as prevalence in the general population. The publication also examined bleeding propensity, considering the severity and frequency of bleeding episodes. It has improved our understanding of VWD, enabling better diagnosis and management by healthcare professionals [24].

## 5. Shear Stress-Induced VWD

Von Willebrand Disease (VWD) is an inherited bleeding disorder caused by a deficiency or dysfunction of von Willebrand factor (VWF), a protein that is essential for blood clotting. AVWFD is a condition in which VWF levels are reduced as a result of an underlying medical condition, such as shear stress-induced von Willebrand disease (SS-VWD).

SS-VWD is a type of AVWFD that occurs as a result of high shear stress on the VWF molecules, which can cause them to be cleaved and degraded. This can occur in the context of AVR procedures, where PPM can result in high shear stress on the artificial valve and an increased risk of AVWFD.

Acquired von Willebrand factor deficiency, in particular type 2A of von Willebrand syndrome, has been described in patients with aortic valve stenosis due to high shear forces developed during passage through the narrowed valve orifice, that determine structural changes in this molecule by exposing the bond between certain amino acids, resulting in proteolysis of the highest-molecular-weight multimers (HMWM) of VWF which are most effective in platelet-mediated hemostasis [18]. Therefore lower levels (<10.5%) of circulation HMWM of VWF increase the risk of bleeding [25]. These days the current method is VWF multimer analysis.

Patients with severe aortic stenosis have been found with levels of HMWM of VWF up to 50% less than the normal value [26].

Patients with aortic stenosis may also be diagnosed with Heyde’s syndrome, a gastrointestinal bleeding complication, due to acquired VWD [27,28].

The severity of acquired VWD is related to the aortic stenosis severity, correlating especially with the maximal pressure gradient rather than the aortic valve area, indicating that proteolysis of HMW multimers of VWF is strongly affected by high shear stresses [28].

Structural abnormalities in VWF show a direct correlation to the transprosthetic pressure gradient; therefore, patients who develop PPM after AVR, which is associated with persistently high shear forces despite normally functioning prosthesis, could determine acquired vWF deficiency.

The clinical presentation of SS-VWD can vary, but it can include symptoms such as bleeding, bruising, and abnormal blood clotting. In some cases, SS-VWD can also lead to thromboembolic events, such as stroke or deep vein thrombosis. In these cases, the etiology of deep venous thrombosis must be differentiated from other causes, such as chronic venous disease [29]. These complications can have a significant impact on patient quality of life and can also increase the risk of morbidity and mortality [30].

Diagnosis of SS-VWD typically involves measuring VWF levels in the blood and assessing the patient’s bleeding history and clinical presentation. VWF levels can be measured using various techniques, including ELISA, immunoturbidimetry, and ristocetin co-factor assays.

In order to provide a clear overview of the relationship between acquired VWD and post-AVR with PPM, we have included a table (see Table 4) summarizing the findings of three key studies on this topic [31,32,33]. These studies suggest a strong correlation between PPM and AVWFD, with the severity of stenosis and the size of the prosthesis playing important roles. However, it should be noted that while the studies included in the table do provide evidence for the relationship between PPM and AVWFD, there is still a need for more research in this area, especially studies with larger sample sizes. In fact, some of the studies included in the table did not specifically examine the relationship between PPM and AVWFD but were still included due to their relevance.

A study conducted by Yoshida et al. in 2006 [31] investigated the effect of aortic valve replacement on von Willebrand factor levels in patients with aortic stenosis. The study included 29 patients with severe AS, among whom 8 reported episodes of bleeding in the 6 months before surgery. Cutaneous or mucosal bleeding were the most common types of bleeding reported, with three patients having a history of major bleeding (cerebrovascular or gastrointestinal hemorrhage). All bleeding episodes occurred in the absence of oral anticoagulant treatment. After surgery, all patients were initiated on oral anticoagulation therapy with warfarin and monitored for anticoagulation with routine prothrombin time and international normalized ratio measurements. One patient with PPM had an episode of cerebrovascular bleeding two months after surgery, despite having a postoperative VWF antigen level within the normal range. The study found that patients with PPM had longer cardiopulmonary bypass time, longer aortic cross-clamp time, and greater mean blood loss than patients without PPM. However, there was no difference in the ratio of valve types used between the two groups. One patient with PPM underwent re-exploration for hemorrhage after aortic valve replacement, and one patient died of pneumonia 30 days postoperatively. Preoperatively, shear-induced platelet aggregation was decreased in all patients with severe AS, but increased in all patients after aortic valve replacement. Preoperative bleeding time was greater than postoperative bleeding time, but postoperative bleeding time was greater in patients with PPM than those without PPM. Preoperative VWF levels were particularly low in patients with bleeding episodes, and postoperative VWF antigen was significantly lower in patients with PPM than in those without PPM, despite postoperative improvement in hemodynamic variables. This suggests that the increased shear stress and primary hemostatic abnormalities related to a decrease in VWF persist in patients with PPM after surgery [31].

Vincentelli et al. (2003) examined 42 patients with severe aortic stenosis who underwent valve replacement and found that platelet-function abnormalities under high shear stress, decreased von Willebrand factor collagen-binding activity and the loss of the largest multimers, or a combination of these, were present in 67 to 92 percent of patients with severe aortic stenosis and correlated significantly with the severity of valve stenosis. The study also found that primary hemostatic abnormalities were completely corrected on the first day after surgery but tended to recur at six months when there was a mismatch between the patient and the prosthesis. Furthermore, the study suggests that type 2A von Willebrand syndrome is common in patients with severe aortic stenosis and that von Willebrand factor abnormalities are directly related to the severity of aortic stenosis and are improved by valve replacement in the absence of mismatch between patient and prosthesis [32]. This information may be particularly relevant to patients with patient-prosthesis mismatch (PPM), as they may be at increased risk of recurrent hemostatic abnormalities post-surgery. However, it should be noted that the study by Vincentelli et al. did not specifically examine the relationship between PPM and von Willebrand factor abnormalities.

According to the study “von Willebrand Factor Abnormalities and Heyde Syndrome in Dysfunctional Heart Valve Prostheses” by Blackshear et al. (2016), acquired abnormalities of VWF multimers are associated with aortic prosthesis dysfunction. The study included a total of 136 patients, and VWF multimers were abnormal in 20 of 24 patients with dysfunctional aortic valve prosthesis, compared to only 1 of 26 patients with normal functioning aortic valve prosthesis. In addition, the severity of VWF dysfunction was also found to be greater in patients with dysfunctional aortic valve prostheses compared to those with normal functioning prostheses. Moreover, the study found that gastrointestinal bleeding was noted in 6 of the 24 patients with aortic prosthesis dysfunction, and gastrointestinal angiodysplasia was noted in five of these patients. This suggests that acquired bleeding may be associated with VWF abnormalities in patients with dysfunctional aortic valve prostheses. Therefore, the study suggests that laboratory testing for VWF abnormalities could add value in quantifying aortic prosthesis dysfunction and may provide an explanation for acquired bleeding in some patients [33].

SS-VWD can also occur in patients after undergoing an aortic valve replacement procedure, even with a normally functioning prosthesis and no criteria for PPM [34]. In a recent case report by Yang et al. (2022), a patient developed AVWFD after undergoing aortic valve replacement with a normally functioning mechanical valve. The patient had a high-output cardiac state which resulted in increased shear stress on the aortic valve and subsequent damage to von Willebrand factor multimers. This case report adds to the growing body of literature suggesting that high shear stress on the aortic valve may be a contributing factor in the development of AVWFD in patients undergoing AVR. Even in the presence of a normally functioning mechanical valve, patients with high-output cardiac states may be at increased risk for AVWFD. These findings suggest that careful management of cardiac abnormalities may be important in preventing the development of AVWFD in this patient population. However, further research is needed to understand better the mechanisms underlying AVWFD.

## 6. Conclusions

Acquired von Willebrand factor deficiency is a rare but potentially serious condition that can occur after an AVR procedure. PPM, which occurs when the size of the prosthetic valve is mismatched with the patient’s native aortic valve, has been shown to be a risk factor for the development of acquired VWF deficiency.

Diagnosing VWF deficiency involves a combination of laboratory testing, clinical history, and physical examination. The specific tests used will depend on the type of VWF deficiency being evaluated, as well as the patient’s symptoms and medical history. Accurate diagnosis is essential for effective treatment and management of VWF deficiency.

Genetic testing for von Willebrand Factor deficiency in patients with severe aortic stenosis can provide valuable information about their bleeding risk and help guide their treatment. It can also help identify patients who may benefit from specialized perioperative management to reduce their risk of bleeding complications during AVR surgery. As such, genetic testing should be considered as part of the routine preoperative evaluation for patients with severe aortic stenosis.

The impact of PPM on long-term survival remains controversial. Severe PPM has been associated with decreased long-term survival in some studies, but other studies have found no significant association. Additional research is needed better to understand the impact of PPM on long-term outcomes and to identify strategies to minimize its occurrence and optimize patient outcomes.

In conclusion, acquired VWF deficiency after AVR procedures should be recognized as a potential complication and should be carefully monitored in patients with PPM. In severe cases, the optimal treatment is re-operation, which has been shown to improve patient outcomes. Further research is needed to understand better the underlying mechanisms of shear stress-induced VWF deficiency and to develop effective strategies for preventing and treating this condition. By increasing our understanding of acquired VWF deficiency, we can work towards improving outcomes for patients undergoing aortic valve replacement procedures and reducing the risk of bleeding complications.

## Figures and Tables

**Table 1:** Aortic valve stenosis grading.

	Aortic Jet Velocity (m/s)	Mean Gradient (mmHG)	Valve Area (cm^2^)
Normal	≤2.0	<5	3.0–4.0
Mild	<3.0	<25	>1.5
Moderate	3.0–4.0	25–40	1.0–1.5
Severe	>4.0	>40	<1.0

**Table 2:** Normal reference values of effective orifice areas for the prosthetic aortic valves [12].

Prosthetic Valve Size (mm)	19	21	23	25	27	29
Stented bioprosthetic valves						
Mosaic	1.1 ± 0.2	1.2 ± 0.3	1.4 ± 0.3	1.7 ± 0.4	1.8 ± 0.4	2.0 ± 0.4
Hancock II	-	1.2 ± 0.2	1.3 ± 0.2	1.5 ± 0.2	1.6 ± 0.2	1.6 ± 0.2
Carpentier-Edwards Perimount	1.1 ± 0.3	1.3 ± 0.3	1.5 ± 0.4	1.8 ± 0.4	2.1 ± 0.4	2.2 ± 0.4
Carpentier-Edwards Magna	1.3 ± 0.3	1.5 ± 0.3	1.8 ± 0.4	2.1 ± 0.5	-	-
Biocor (Epic)	1.0 ± 0.3	1.3 ± 0.5	1.4 ± 0.5	1.9 ± 0.7	-	-
Mitroflow	1.1 ± 0.2	1.2 ± 0.3	1.4 ± 0.3	1.6 ± 0.3	1.8 ± 0.3	-
Trifecta	1.4	1.6	1.8	2.0	2.2	2.4
Stentless bioprosthetic valves						
Medtronic Freestyle	1.2 ± 0.2	1.4 ± 0.2	1.5 ± 0.3	2.0 ± 0.4	2.3 ± 0.5	-
St Jude Medical Toronto SPV	-	1.3 ± 0.3	1.5 ± 0.5	1.7 ± 0.8	2.1 ± 0.7	2.7 ± 1.0
Prima Edwards	-	1.3 ± 0.3	1.6 ± 0.3	1.9 ± 0.4	-	-
Mechanical valves						
Medtronic-Hall	1.2 ± 0.2	1.3 ± 0.2	-	-	-	-
St Jude Medical Standard	1.0 ± 0.2	1.4 ± 0.2	1.5 ± 0.5	2.1 ± 0.4	2.7 ± 0.6	3.2 ± 0.3
St Jude Medical Regent						
Carbomedics Standard and Top Hat	1.0 ± 0.4	1.5 ± 0.3	1.7 ± 0.3	2.0 ± 0.4	2.5 ± 0.4	2.6 ± 0.4

**Table 3:** VWD: inheritance, prevalence and bleeding propensity. Modified and adapted from [24].

Type	Inheritance	Prevalence	Bleeding Propensity
Type 1	Autosomal dominant	≤1%	Mild to moderate
Type 2A	Autosomal dominant/recessive	Uncommon	Variable-moderate
Type 2B	Autosomal dominant	Uncommon	Variable-moderate
Type 2M	Autosomal dominant/recessive	Uncommon	Variable-moderate
Type 2N	Autosomal recessive	Uncommon	Variable-moderate
Type 3	Autosomal recessive	Rare	High

**Table 4:** Relationship PPM–AVWFD.

Study	Number of Patients	Diagnosis of AVWFD	Diagnosis of PPM	Relationship between AVWFD and PPM
Vincentelli et al. [32]	*n* = 42, AVR for AS, 10 PPM	VWF antigen, VWF activity, FVIII coagulant activity	Echocardiography	Suggesting a positive correlation between AVWFD and PPM
Yoshida et al. [31]	*n* = 29, AVR for AS	Platelet aggregation, VWF multimeric analysis	Echocardiography	Suggesting a positive correlation between AVWFD and PPM
Blackshear et al. [33]	*n* = 136 VR, out of which 26 AVR normal functioning, 24 AVR malfunctioning	VWF antigen, VWF activity and quality loss of HMWM	Echocardiography	Suggesting a positive correlation between AVWFD and prosthesis malfunction

## Data Availability

Data sharing not applicable. No new data were created or analyzed in this review article.
